# Several Proinflammatory Genes’ Variability and Phenotypes of Atopic Dermatitis in Czech Adult AD Patients

**DOI:** 10.3390/genes16060703

**Published:** 2025-06-12

**Authors:** Vladimír Vašků, Anna Vašků

**Affiliations:** 1 1st Department of Dermatology, St. Ann’s Faculty Hospital, Faculty of Medicine, Masaryk University, 60200 Brno, Czech Republic; 2 Department of Pathophysiology, Faculty of Medicine, Masaryk University, 62500 Brno, Czech Republic

**Keywords:** atopic dermatitis, genetics, marker, phenotype, family history

## Abstract

Background: The etiopathogenesis of atopic dermatitis is complicated, and it includes aspects such as dysfunction of the skin barrier, changes in immune responses, IgE-mediated hypersensitivity, and many characteristics of the environment. Regarding skin barrier dysfunction, a number of genetic changes have been described. This genetic predisposition could be related to the phenotypes of atopic dermatitis. Aim: In this study, several polymorphisms in five proinflammatory genes were associated with certain phenotypes of AD patients (genotype–phenotype study). Methods: In total, 89 unrelated AD Czech (Caucasian) patients were genotyped regarding five proinflammatory gene polymorphisms (angiotensinogen AGT M235T, AGT-6 G/A, TNF-α-238 G/A, TNF-β Fok1, IL-6-174 C/G and IL-6-596 G/A). Genotyping was performed using PCR and restriction analysis. For phenotypes, patients’ sex, age and personal and family history of atopy, aero- and food allergies and other complex diseases were evaluated. Results: A significant association with transepidermal water loss (TEWL) measured on the forearm was found with the AGT M235T polymorphism (*p* = 0.02). For the AG genotype of TNF-α-238 G/A, a six-times higher risk for a family history of diabetes mellitus compared to other examined aspects of family history was found (*p* = 0.02). A family history of thyreopathy was associated with the IL-6-174 G/C polymorphism when compared to a family history of other complex diseases. The GG genotype had a ten-times higher risk for a family history of thyreopathy compared to the other genotypes (*p* = 0.004). This result was highly specific (0.914). The GG genotype of IL-6-596 G/A was associated with a family history of thyreopathy, with the same result (*p* = 0.004). Moreover, the G allele of IL-6-174 G/C was associated with a family history of thyreopathy compared to AD patients without a positive family history of complex diseases (*p* = 0.03). In AD men, the MM genotype of the AGT M235T gene was found to be associated with food allergies (*p* = 0.004). This result was highly sensitive (0.833). A family history of cardiovascular disease in AD men was associated with AGT-6 G/A variability. The A allele was found to be six times more frequent in patients with a positive family history of cardiovascular disease (*p* = 0.02, with high sensitivity and specificity (0.700 and 0.735, respectively)). A family history of diabetes mellitus was associated with the TNF-β Fok1 polymorphism, where the B1 allele was almost six times more frequent in AD men with a positive family history of diabetes mellitus (*p* = 0.02), with high sensitivity (0.85). A significant association between TEWL measured on the forearm and the AGT M235T polymorphism was found when AD women were carriers of the MM genotype, with a median of 25 and range 4–61; those patients with the MT genotype had a median of 10 and range of 0.3–39; and patients with the TT genotype had a median of 5 and range of 3–40, *p* = 0.003. The polymorphism AGT-6 G/A was associated with different ages of eczema onset. The AG genotype was almost nine times more risky for the youngest group (0–7 years) compared to the oldest group (more than 18 years) (*p* = 0.02), with high specificity for this result. Conclusions: Our results in the field of cytokine signaling in the immune system in patients with atopic dermatitis are in agreement with those of GWASs. We suggest that cost-effective and simple PCR tests may be the best approach for the rapid and optimal collection of valid genetic information in clinical practice.

## 1. Introduction

Atopic dermatitis (AD) is defined as a chronic inflammatory skin disease with largely heterogeneous phenotypes. Typically, it develops under environmental stimuli in persons with a genetic predisposition to the disease. Dysregulation of innate as well as adaptive immunity seems to play a substantial role, leading to the clinical manifestation of AD [1].

AD is characterized by pruritus, which is often more intense at night, and by dry and indurated skin covered by itching papules. Their occurrence leads to scratching, where the release of a clear fluid may be observed [1]. In AD patients, the consequent development of atopic disorders can be expected, which include asthma, allergic rhinitis and food allergies [2]. Patients with AD are prone to infections because pathogen recognition and control of epidermal permeability for pathogens are impaired due to the dysfunction and damage of the skin barrier [3].

In most individuals (80%), the clinical manifestation of AD occurs during the first few years of life [4]. Approximately 60% of patients will enter remission in adolescence [5]. The disease symptoms are different among different age groups and skin types [6]. At present, more well-performed epidemiological studies on childhood and adulthood AD are needed, because the incidence of AD during adulthood is not documented conclusively [7].

Quality of life is strongly influenced by atopic dermatitis. The psychosocial load of atopic dermatitis (AD) is highly stressful. Intense itching, scratching, disturbances of sleep, lifestyle limitations and restricted activities can be identified. Moreover, adults with AD have an increased risk for the development of anxiety and depression compared to individuals without AD [8].

Alexithymia is a psychological construct describing difficulties in understanding and describing one’s own emotions. Moreover, the differentiation of feelings from bodily signals of arousal is affected. In the general population, alexithymia’s prevalence is approximately 10% [9]. An increased frequency of alexithymia has been described in many dermatological diseases, including atopic dermatitis. It was found to be more frequent in severe AD patients (43.6%) compared to mild AD patients (15.6%). Alexithymia is correlated with the intensity of itching and with disturbances of sleep. The disease severity was found to be a predictor of alexithymia [10].

Keratinocytes, stromal and endothelial cells, Langerhans cells, macrophages and other immune cells in the skin have pattern recognition receptors (PRRs), which serve as initiators of epidermal immune reactions through the recognition of pathogenic patterns known as PAMPs. PRRs are responsible for pathogen recognition by activating the innate immune system and antigen-specific adaptive immunity [11].

PRRs represent several families of receptors, such as Toll-like receptors (TLRs). The activation of their signaling pathways leads to the activation of many transcription factors. They regulate the transcription of hundreds of genes with proinflammatory and/or anti-inflammatory functions. Inflammatory cytokines, together with antimicrobial peptides, are able to develop rapid reactions against pathogens. TLRs are able to modify the response of adaptive immunity by supporting the maturation of dendritic cells, as well as the function of T and B cells [12].

In keratinocytes, Toll-like receptors (TLR 2 and TLR 3) are the major PRRs. After bacterial lipopeptides’ recognition by TLR 2, IL-6 and tumor necrosis factor-α (TNF-α) production is provoked. TNF-α, alone or in combination with other cytokines, such as IL-4 or IL-13 (Th2 profile), can decrease the lengths of long-chain free fatty acids and ceramides, which supports alterations to the structure and function of the skin barrier [13]. In dermal fibroblasts, other TLRs, such as TLRs 2 and 4, can be identified. They recognize bacterial lipopeptides and fungal pathogens and also support the production of proinflammatory cytokines [14].

Inflammatory cytokines, such as IL-6 and TNF-α, stimulate neighboring cells to produce chemokines and adhesion molecules, which will recruit other immune cells to the site of invasion. TLRs can identify both damage-associated molecular patterns (DAMPs), expressed by damaged skin cells, and pathogen-associated molecular patterns (PAMPs), expressed by invading pathogens. Such recognition will stimulate both innate and acquired immunity and will enhance the production of proinflammatory cytokines. However, excessive TLR activation can lead to T-lymphocyte-mediated autoimmunity, further predisposing individuals to chronic inflammation and adverse skin conditions [15].

The presence of an association between thyroid autoimmunity and atopic dermatitis has been described. Thus, autoreactivity might participate in the pathogenesis of AD [16]. In fact, other lymphocyte subsets, such as Th17 cells and regulatory T cells (Tregs), have been identified as a common link between atopy and autoimmunity. Thyroid autoantibodies, especially TgAb, seem to have a significant influence in allergic disorders. Screening for thyroid function and autoimmunity is clinically useful to monitor the clinical progress of patients with allergic symptoms [17].

TNF-α is a 26 kDa transmembrane protein that is released into the circulation as a 17 kDa soluble protein after extracellular cleavage by a metal proteinase (88). TNF-α, produced in the adipose tissue, is known to represent a molecular link between obesity and insulin resistance [18]. In the adipose tissue, TNF-α stimulates lipolysis by modifying the expression of genes involved in adipogenesis and lipogenesis using NF-κB. This leads to elevations in the levels of fatty acids in the blood. Macrophages in the adipose tissue produce nearly all TNF-α. The TNF-α mRNA concentrations are increased in the adipose tissue of obese individuals.

In the liver, TNF-α increases the expression of genes involved in fatty acid synthesis de novo. At the same time, a decrease in the expression of genes responsible for fatty acid oxidation is supported by TNF-α. TNF-α impairs insulin signaling through the serine phosphorylation of both the insulin receptor and insulin receptor substrate 1 (IRS-1). Both these effects can lead to the decreased activation of phosphoinositol-3-kinase, responsible for many metabolic effects of insulin. The TNF-α concentrations in the blood rise with increasing obesity and correlate with insulin resistance.

Interleukin-6 (IL-6) is a protein of 22–27 kDa, with various degrees of glycosylation. The human adipose tissue produces substantial amounts of IL-6; this secretion represents 10–30% of the IL-6 circulating levels. IL-6 plays a direct role in insulin resistance by altering insulin signaling in hepatocytes. This effect is mediated by the induction of suppressor of cytokine signaling-3 (SOCS-3), which inhibits insulin-dependent insulin receptor autophosphorylation. Plasma IL-6 is highly correlated with body mass and inversely related to insulin sensitivity [19].

TNF-α, singly or in combination with Th2 cytokines, modulates the states of lipids in the epidermal barrier in AD lesional skin, which supports the inflammatory contribution of TNF-α during the development of atopic dermatitis. Moreover, TNF-α, singly or in combination with Th2 cytokines, changes the proportion of saturated FFAs in the skin [15].

IL-6 signaling is complex. Systemic inflammation can be expected, resulting from IL-6’s effects on the liver (acute-phase reaction) and endothelium. Elevated IL-6 levels in obesity and central IL-6 resistance must be taken in account. About one-third of the total circulating level of IL-6 is formed in adipocytes [20]. Waist circumference is mainly correlated with the plasma IL-6 and CRP levels, highlighting the crucial role of visceral adiposity in inflammation [19]. In addition, it promotes the expression of adhesion molecules on endothelial cells for leucocytes, enhancing vascular damage and inflammation [21,22].

Circulating IL-6 is the most important factor regulating the hepatic acute-phase response. Therefore, IL-6 seems to be a mediator of proinflammatory signaling from the adipose tissue [19].

A significant positive correlation between blood pressure and the circulating levels of angiotensinogen (AGT) has been found. AGT is mainly produced by the liver. The adipose tissue is considered a major extrahepatic source of AGT. Again, the higher production of angiotensinogen (similarly to that of TNF-α and IL-6) in the adipose tissue can contribute to increased circulating levels of ATG in obese individuals [19].

All cytokines exhibit interindividual variability. Therefore, the aim of our study was to examine the possible associations of several polymorphisms in the angiotensinogen, TNF and IL-6 genes with certain phenotypes in AD patients (a genotype–phenotype study).

## 2. Methods

### 2.1. Subjects

In total, 89 unrelated AD Czech (Caucasian) patients were enrolled in this study; 58 were men, with a median age of 28 years and a range of 18–58 years, and 31 were women, with a median age of 26 years and a range of 18–71 years. In 65 (73%) patients, an elevation in IgE was diagnosed. Regarding other phenotypes, aspects related to the family and personal history of atopy, aero- and food allergies and other complex diseases were evaluated (Table 1 and Table 2).

### 2.2. Laboratory Methods

All AD patients included in the study were genotyped according to five proinflammatory gene polymorphisms (angiotensinogen AGT M235T, AGT-6 G/A, TNF-α- 238 G/A, TNF-β Fok1, IL-6-174 C/G and IL-6-596 G/A). Genomic DNA was isolated from peripheral leukocytes by a standard technique using proteinase K. Genotyping for the polymorphisms was performed by PCR with restriction analysis according to standardized protocols published previously [23,24].

### 2.3. Statistics

Using χ^2^ tests, the distributions of genotype and allelic frequencies, as well as the consistency of genotype frequencies, were calculated, as well as the Hardy–Weinberg equilibrium. The odds ratio (OR) and 95% confidence interval were calculated to estimate the higher risks related to the genotypes/alleles. To calculate the significance of the OR, Fisher’s exact test was used. Clinical Calculator 1 from VassarStats was applied for the calculation of the sensitivity and specificity of the results. The program Statistica v. 14.0 (Statsoft Inc., Tulsa, OK, USA) was used.

## 3. Results

### 3.1. All AD Patients

A significant association between TEWL measured on the forearm and the AGT M235T (rs699) polymorphism was found. The carriers of the MM genotype had a median of 18 g/m^2^/h, with a range of 4–61 g/m^2^/h; the patients with the MT genotype achieved 10 g/m^2^/h, with a range of 0.3–39 g/m^2^/h; and patients with the TT genotype had 7 g/m^2^/h, with a range of 3–40 g/m^2^/h, *p* = 0.02.

For the AG genotype of TNF-α-238 G/A (rs361525), a higher risk for a family history of diabetes mellitus compared to other examined family histories was found [OR = 6.48, 95% confidential interval (CI) 0.7–59.6, *p* = 0.02, sensitivity 0.07, specificity 1.0, power test 0.289].

A family history of thyreopathy was associated with Il-6-174 C/G (rs1800795) when compared to a family history of other complex diseases. In this case, the GG genotype had a 10-times higher risk for a family history of thyreopathy compared to the other genotypes [OR = 10.6, 95% CI 2.27–49.54, *p* = 0.004, sensitivity 0.5, specificity 0.914, power test 0.766]. The GG genotype of IL-6-596 G/A (rs1800797) was associated with a family history of thyreopathy, with the same results [OR = 10.6, 95% CI 2.27–49.54, *p* = 0.004, sensitivity 0.5, specificity 0.914, power test 0.766]. Moreover, the G allele was associated with a family history of thyreopathy compared to AD patients without a positive family history of complex diseases [OR = 3.34, 95% CI 1.10–10.19, *p* = 0.03, sensitivity 0.65, specificity 0.64, power test 0.473].

When the patients were divided according to sex, another significant association with variability in proinflammatory genes was observed.

### 3.2. AD Men

In AD men, the MM genotype of the AGT M235T gene was associated with food allergies [OR = 6.58, 95% CI 1.66–26.04, *p* = 0.004, sensitivity 0.833, specificity 0.568, power test 0.780; Table 3].

A family history of cardiovascular diseases in AD men was associated with AGT-6 G/A variability. The A allele was more frequent in those patients with a positive family history of cardiovascular diseases [OR = 6.48, 95% CI 1.37–30.61, *p* = 0.02, sensitivity 0.7, specificity 0.735, power test 0.589].

A family history of diabetes mellitus was associated with the TNF-β Fok1 polymorphism, where the B1 allele was more frequent in AD men with a positive family history of diabetes mellitus [OR = 5.667, 95% CI 1.30–24.53, *p* = 0.02, sensitivity 0.85, specificity 0.50, power test 0.570].

### 3.3. AD Women

A significant association between TEWL measured on the forearm and the AGT M235T polymorphism was found, where AD women—carriers of the MM genotype—had a median 25 g/m^2^/h and a range of 4–61 g/m^2^/h; female patients with the MT genotype had 10 g/m^2^/h, with a range of 0.3–39 g/m^2^/h; and female patients with the TT genotype had 5 g/m^2^/h, with a range of 3–40 g/m^2^/h, *p* = 0.003; see Table 4.

The polymorphism AGT-6 G/A was associated with different ages of eczema onset. The AG genotype was a risk for the youngest group (0–7 years) compared to the oldest group (more than 18 years) [OR = 8.87, 95% CI 1.05–74.95, *p* = 0.02, sensitivity 0.425, specificity 0.923, power test 0.503; Table 5].

The GG genotype of the IL-6-174 C/G polymorphism was associated with a positive family history of atopy [OR = 6.67, 95% CI 1.36–32.70, *p* = 0.02, sensitivity 0.930, specificity 0.333, power test 0.550; Table 6]. Similar results were obtained for the GG genotype of IL-6-596 A/G.

## 4. Discussion

A meta-analysis of 1,094,060 individuals (37,541 cases; 1,056,519 controls) detected 77 loci associated with atopic dermatitis. They were identified mostly in intronic or intergenic genomic regions [25].

AD susceptibility was found to be supported by a high contribution of genetic variability. Heritability estimates were approximately 75% in twin studies [26]. Previous GWASs have identified approximately 80 genetic risk loci that explain about 30% of the variation in AD susceptibility [27,28,29].

The major predisposing genetic factors include loss-of-function variants in the *FLG* gene. FLG is essential in maintaining epidermal homeostasis, and *FLG* loss-of-function variants lead to skin barrier dysfunction, which can contribute to dry skin development and the worsening of the immunological properties of the skin, leading to a dysfunctional/damaged skin barrier [30,31]. Further risk loci contributing to epidermal resistance and integrity include variants in *DSC1* and *SERPINB7* [32]. Variants in multiple immune-regulatory genes, such as *IL13* and *IL6R*, have been linked to immune dysregulation in AD.

Polymorphisms of some immunologically active pathway genes are associated with an increased risk of AD, particularly through alternations in the T-helper (Th) type 2 signaling pathway. The upregulation of interleukin (IL)-4 and IL-13 lowers filaggrin expression, which leads to skin barrier defects. Other immune-related genes that can contribute to the genetic predisposition to AD include IL-4R, IL-13R, IL-31, IL-33, signal transducer and activator of transcription (STAT) 6, thymic stromal lymphopoietin (TSLP) and its receptors (IL-7R and TSLPR), interferon regulatory factor 2, Toll-like receptor 2 and the high-affinity IgE receptor (FcεRI) α gene in specific populations [33].

The largest AD GWAS to date (discovery N = 1,086,394) was performed in 2023. In total, 81 loci in the European-only analysis and 10 additional loci in the multi-ancestry analysis were identified. The implicated genes were predominantly in immune pathways of relevance to inflammation in atopic patients [29]. Among them, rs41293876 in TNF was discovered.

It is necessary to take into account other mechanisms that can influence the transcriptional and translational processing of genes/proteins. Some epigenetic mechanisms can change the transcription of genes coding for key molecules for the skin barrier [33]. Using discovery proteomics, more than 1000 proteins were identified, some of them with posttranslational changes (citrullination) [34].

According to our results, a family history of thyreopathy was associated with IL-6 polymorphisms. Another IL-6 gene promoter (−572 C/G) polymorphism has been described to be a specific genetic marker for susceptibility to Hashimoto’s thyroiditis [35].

The polygenic risk score (PRS) approach seeks to summarize the risks of individual genetic variants weighted by their disease-specific effect sizes, in which these effect sizes are typically derived from strongly evaluated genome-wide association studies (GWASs) [36]. This is a promising approach for the near future.

Our results in the field of cytokine signaling in the immune system in patients with atopic dermatitis seem to be agreement with GWASs. Most of our results provide new information about potential proinflammatory genetic markers with high sensitivity and/or specificity. A limitation of this study is the relatively small number of patients, which is reflected in the lower power test values of the results. A larger sample of adult patients with atopic dermatitis should be tested in a Central European population to confirm our results. Finally, we suggest that cost-effective and simple PCR tests may be the optimal and most valuable approach to the introduction of valid genetic information in dermatological practice.

## Figures and Tables

**Table 1:** Family history of diseases.

AD Patients M/W (58/31)	N = 89	%
Atopy	64	72
Psoriasis	12	13
Cardiovascular diseases	23	26
Diabetes mellitus	27	30
Thyreopathy	11	12

**Table 2:** Personal history of diseases.

AD Patients M/W (58/31)	N = 89	%
Atopy	48	54
Food allergy	33	37
Aero-allergy	70	79
Gastrointestinal diseases	7	8
Cardiovascular diseases	11	12
Diabetes mellitus	2	2
Thyreopathy	4	4

**Table 3:** Food allergy and M235T ATG polymorphism in AD men.

Food Allergy AD Men (N = 31)	ATG 235 MM	ATG 235 TT	ATG 235 MT	Rows
Yes	6 (67%)	0 (%)	3 (33%)	9 (29%)
No	5 (23%)	8 (36%)	9 (41%)	22 (71%)
All	11 (35%)	8 (26%)	12 (39%)	31 (100%)

**Table 4:** Transepidermal water loss (TEWL) on forearm and AGT M235T polymorphism.

Genotype ATG M235T AD Women (N = 58)	TEWL Forearm N	TEWL Forearm Median g/m^2^/h	TEWL Forearm Minimal g/m^2^/h	TEWL Forearm Maximal g/m^2^/h
MM	12	25.15	4.00	60.90
MT	35	10.10	0.30	39.10
TT	11	5.40	3.10	40.30
All	58	10.85	0.30	60.90

**Table 5:** Age group at onset of eczema and AGT-6 A/G.

Onset of Eczema Age Group AD Women (N = 58)	ATG-6 GG	ATG-6 AG	ATG-6 AA	Rows
0–7 years	14 (35%)	17 (43%)	9 (22%)	40 (69%)
8–18 years	3 (60%)	2% (40%)	0 (0%)	5 (9%)
More than 18 years	9 (69%)	1 (8%)	3 (23%)	13 (22%)
All	26 (45%)	20 (34%)	12 (21%)	58 (100%)

**Table 6:** Family history of atopy and IL-6-174 C/G.

Family History of Atopy AD Women	IL-6 174 GG	IL-6 174 CC	IL-6 174 GC	Rows
Yes	3 (6%)	20 (47%)	20 (47%)	43 (74%)
No	5 (33%)	5 (33%	5 (33%)	15 (26%)
All	8 (14%)	25 (43%)	25 (43%)	58 (100%)

## Data Availability

The original contributions presented in this study are included in the article. Further inquiries can be directed to the corresponding authors.
